# Eye tracking in developmental cognitive neuroscience – The good, the bad and the ugly

**DOI:** 10.1016/j.dcn.2019.100710

**Published:** 2019-09-27

**Authors:** Roy S. Hessels, Ignace T.C. Hooge

**Affiliations:** a Experimental Psychology, Helmholtz Institute, Utrecht University, Utrecht, The Netherlands; b Developmental Psychology, Utrecht University, Utrecht, The Netherlands

**Keywords:** Eye tracking, Data quality, Development, Data analysis, Longitudinal

## Abstract

Eye tracking is a popular research tool in developmental cognitive neuroscience for studying the development of perceptual and cognitive processes. However, eye tracking in the context of development is also challenging. In this paper, we ask how knowledge on eye-tracking data quality can be used to improve eye-tracking recordings and analyses in longitudinal research so that valid conclusions about child development may be drawn. We answer this question by adopting the data-quality perspective and surveying the eye-tracking setup, training protocols, and data analysis of the YOUth study (investigating neurocognitive development of 6000 children). We first show how our eye-tracking setup has been optimized for recording high-quality eye-tracking data. Second, we show that eye-tracking data quality can be operator-dependent even after a thorough training protocol. Finally, we report distributions of eye-tracking data quality measures for four age groups (5 months, 10 months, 3 years, and 9 years), based on 1531 recordings. We end with advice for (prospective) developmental eye-tracking researchers and generalizations to other methodologies.

## 1 Introduction

What wonder it must be to see the world through a child's eyes as they grow up. While it is physically impossible to see the world through a child's eyes, it illustrates the intuitive appeal of investigating one's looking behavior. Indeed, researchers have been interested in the looking behavior of children for a long time (Aslin, 2007). Through eye tracking, gaze location can be objectively measured from children as young as a few days old, and up to adulthood. As such, eye tracking has been one of the main research methods in the last decades for gaining insights into early (neuro)cognitive development (see e.g. Aslin and McMurray, 2004, Aslin, 2012, Oakes, 2012). Eye tracking is therefore also one of the main methods used in the YOUth study 1 investigating individual developmental trajectories. Although eye tracking is often hailed as an excellent tool for studying early development (*the good*), eye tracking in the context of development is also challenging. For one, young children cannot be instructed to behave to the experimenter's wishes. Moreover, the quality of eye-tracking data obtained in infant research is often low compared with the quality of eye-tracking data obtained in adult research. If the analysis tools used are susceptible to differences in data quality, invalid conclusions may be drawn about child development, particularly at the individual level (*the bad*). In this paper, we adopt the data-quality perspective and use this to scrutinize the entire procedure of eye-tracking research. We thus address *the ugly* problems in eye-tracking research that may not always be the primary interest when an experimental study is conceived, yet which need be solved to ensure that valid conclusions about development can be drawn.

This goal of this paper is two-fold. First, we give a brief overview of the advantages and disadvantages of using eye tracking in developmental research. While the advantages may be intuitively clear, many of the disadvantages have been discussed mainly in the methodological, not the developmental, eye-tracking literature. Hereafter, we answer three outstanding questions with regard to eye-tracking data quality in developmental eye-tracking research. This paper is aimed at the entire spectrum of eye-tracking researchers in developmental cognitive neuroscience or psychology. For example, developmental researchers who are one of the first in their group to conduct eye-tracking research may find helpful suggestions and advice on the important trade offs for experimental setup, design, eye-tracking data collection and analysis. Likewise, experienced researchers may also find inspiration or helpful suggestions for their eye-tracking operation that have hitherto not been discussed in the literature.

### 1.1 The good

Humans rely greatly on vision, and gaze direction and eye movements are coupled to a host of perceptual and cognitive processes. Fast eye movements that redirect the line of sight (saccades) allow the visual world to be sampled (Kowler, 2011), and visual information can thus be gathered for e.g. motor action (Land and Furneaux, 1997) or information-processing tasks such as visual search and reading (Rayner, 1998). Under the common assumptions that where one looks is where one's attention is allocated and that visual information around the gaze location is processed (e.g. Hessels et al., 2019), knowing where children look at a given point in time can thus provide crucial insights into perceptual and cognitive development. Eye tracking has thus been a popular research tool in developmental research. Researchers have, for example, investigated how visual information selection occurs in infants (Amso and Johnson, 2006, Hessels et al., 2016a), or how infants explore faces (Hunnius and Geuze, 2004) and their visual environment (Franchak et al., 2011), among various other topics. The many eye-tracking studies of the past decades have culminated in models on how infants learn about the world (Johnson, 2010), on early developmental trajectories of Autism (Elsabbagh and Johnson, 2016), and even hypotheses about biological niche construction through eye movements (Constantino et al., 2017).

Importantly, eye trackers allow one to obtain a gaze location objectively and non-invasively. Most modern video-based eye trackers give as output a gaze signal (often a location on a screen), which is obtained by filming the eyes of the participant. By determining the locations of the pupil center in the camera image and the centers of reflections of one or several (near-)infrared illuminators, gaze location can be inferred. All that is needed for this is a calibration procedure during which participants look at several known locations in the world (e.g. on a computer monitor) and having the participant sit relatively still. Eye tracking in the context of development has quite a long history. Already halfway through the 20th century, estimations of infants’ gaze location were used to learn about perceptual development (Salapatek and Kessen, 1966). However, this often came at the cost of long hours of manual coding. Nowadays, eye trackers can automatically report gaze location up to a thousand times per second with no manual labor involved at all (for comprehensive work on video-based eye tracking, see Holmqvist et al., 2011). One can even obtain an infant's gaze location online and use this to manipulate the visual stimulus (gaze-contingent eye tracking).

### 1.2 The bad

While the advantages of eye tracking for developmental research seem clear, less attention has been paid to the disadvantages. Among the disadvantages that have been noted, Gredebäck et al. (2009) mention that eye trackers are costly, more so than traditional observational methods for establishing looking times. However, a salary for researchers (or research assistants) to manually code looking behavior may quickly offset the initial cost of an eye tracker. A likely more prominent disadvantage is that eye trackers deliver gaze data as time series (horizontal and vertical components of gaze location over time), which requires specific skills to analyze (e.g. signal processing). More recently, the problems specific to collecting eye-tracking data in developmental research and analyzing these have been addressed. In order to make these problems intelligible, it is crucial that we first clarify what we mean when we refer to the quality of eye-tracking data and the ‘data-quality perspective’.

#### 1.2.1 Eye-tracking data quality and the data-quality perspective

The quality of eye-tracking data is often characterized by quantifying the accuracy, precision and data loss. *Accuracy* refers to the error between the gaze location reported by the eye tracker and the true gaze location. It may be referred to as the systematic error, and corresponds best to the term ‘validity’ often used in the context of psychological research. It is often operationalized as the difference between a gaze location measured by the eye tracker and an instructed gaze location to the participant. The higher the systematic error, the lower the accuracy. *Precision* refers to the reproducibility of a gaze location by the eye tracker under the assumption that the true gaze location does not vary. It may be referred to as the variable error, and corresponds best to the term ‘reliability’ as commonly used in psychological research. It is often operationalized as the root mean square of the sample-to-sample deviation in gaze location when a participant is assumed to fixate the same location. The higher the sample-to-sample deviation, the lower the precision. Finally, *data loss* refers to the relation between the expected number of measurements of gaze location to be delivered by the eye tracker and the actual number delivered. If, for example, a participant turns their head away from the screen or blinks, the eye tracker cannot report a gaze location. This then constitutes data loss. Data loss can also occur, however, when the participant is looking toward the screen and has their eyes open. This is often considered to be due to ‘technical difficulties’ on the side of the eye tracker.

Generally, researchers in developmental psychology or cognitive neuroscience use an eye-tracker to answer questions from the field of psychology. For example, what can eye-tracking teach us about child development? In our experience, the eye tracker is often taken for granted. Here, we approach eye-tracking research from the data-quality perspective. With this, we mean that we look at eye-tracking data collection and analysis purely from the viewpoint that many aspects of the eye-tracking operation can have a positive or negative effect on data quality. For example, an eye-tracking recording with an infant that moves a lot may yield unusable eye-tracking data. Obviously, this is not desirable. High eye-tracking data quality provides the foundation for solid developmental eye-tracking research, and we believe that it is here that much may be gained. We briefly review the current literature on eye-tracking data quality in developmental research, and highlight which aspects of the eye-tracking operation have already been shown to positively or negatively affect data quality.

#### 1.2.2 Eye-tracking data quality in developmental research

**Fig. 1:**
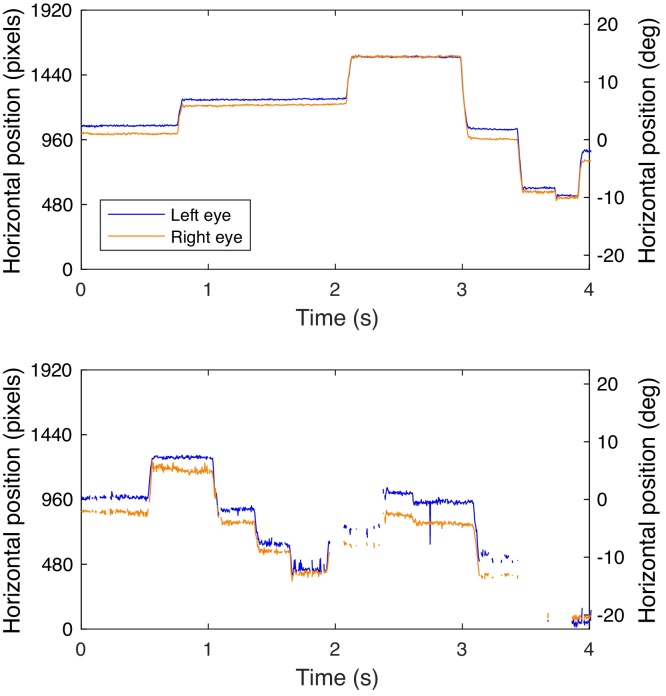
Example horizontal gaze position data for two 10-month-old infants from Hessels et al. (2016a). Gaze positions recorded from the two eyes are given by separate lines (blue for left eye, orange for right eye). *Top:* Example 4-s horizontal gaze position on screen in pixels (left axis) and degrees (right axis) of relatively high quality. *Bottom:* Example 4-s horizontal gaze position on screen in pixels (left axis) and degrees (right axis) of relatively low quality. Degrees are reported under the assumption that the participant's eyes were 65 cm from the screen, and that all areas of the screen were at equal distance from the eyes. (For interpretation of the references to color in this figure legend, the reader is referred to the web version of this article.)

An important question in the context of eye-tracking data quality is which eye tracker to use. Eye-tracker manufacturers generally specify how precise and accurate measurements of gaze location are with their eye trackers. One might thus think that the eye tracker with the best specifications is the one to use in (developmental) research. Yet, these specifications are generally achieved under ‘optimal’ conditions. Optimal refers to recordings with adult participants, who can be well instructed and positioned in a chin rest. Manufacturer specifications are not necessarily representative for situations when participants are unrestrained (Hessels et al., 2015b, Niehorster et al., 2018), as is often the case with infants and toddlers. Indeed, the accuracy and precision of eye-tracking data collected from infants and toddlers are often lower than the accuracy and precision of eye-tracking data obtained from adults (Dalrymple et al., 2018, Hessels et al., 2016b, Hooge et al., 2018b). Notably, lower precision (higher variable errors) in infant eye-tracking data *cannot* be attributed to gaze behavior of the child itself (for example due to infants having lower fixation stability; Seemiller, 2018). Furthermore, many short periods of data loss are observed in infant eye-tracking research, which cannot be attributed to participants looking away or blinking. The interested reader is referred to Wass et al. (2014, p. 5) and Hessels et al. (2015a, p. 5) for more elaborate discussions on the nature of this data loss. Fig. 1 depicts two example gaze-position signals of relatively high and low quality. As can be seen from the top panel, there are clear periods in which the gaze position does not change much (the fixations^2^), and swift changes in gaze position (the saccades). In the example in the Bottom panel of Fig. 1, the precision is much lower, which is evident from the fact that the sample-to-sample difference in gaze position is larger than in the Top panel. Moreover, there are short periods of data loss between 2 and 2.5 s, and larger periods of data loss between 3 and 4 s. These differences are qualified by the statement that the eye-tracking data in the Top panel are of higher quality than the eye-tracking data in the Bottom panel.

Low eye-tracking data quality – defined as low accuracy and precision, and the occurrence of periods of data loss – poses specific problems for data analysis. For example, the number of fixations classified in eye-tracking data and the corresponding average fixation duration may depend on the precision (variable error) of the gaze-position signal (Wass et al., 2014, Holmqvist et al., 2012). More importantly, whether a lower or higher number of fixations are classified in eye-tracking data for lower-precision eye-tracking data depends on the specific fixation-classification algorithm that is used (Hessels et al., 2017). In other words, important measures of gaze behavior (number of fixations and average fixation duration) may be biased by the quality of the eye-tracking data, and in an idiosyncratic way based on the analysis tools used. In an extreme example, Shic et al. (2008) show that the settings used in a fixation-classification algorithm can reverse differences between typically developing children and children with Autism. Clearly, this is an undesirable situation, as conclusions are often drawn about typical and atypical development based on eye-tracking data.

One crucial problem for eye-tracking researchers in developmental research is that most state-of-the-art fixation-classification algorithms are not developed for, or tested on, eye-tracking data of low quality (Hessels et al., 2017). As such, some developmental researchers have even turned to manual correction of fixation classifications (Saez de Urabain et al., 2015), which may lead to biases based on who corrects the classifications (Hooge et al., 2018b). Wass et al. (2013) were the first to develop a fixation-classification algorithm that works with low quality eye-tracking data, using a common algorithm with a set of exclusion rules. However, these exclusion rules can cause a lot of eye-tracking data to be excluded (Hessels et al., 2017). This may not be desirable if there are many children for which this occurs, and if eye-tracking data quality is related in some way to other behavioral characteristics. In the latter case, conclusions may be drawn about perceptual or cognitive development based on a biased sample of children. Recently, new fixation-classification algorithms have been developed (Renswoude et al., 2018, Hessels et al., 2017) that can work with eye-tracking data within a larger range of variable error and data loss. However, much is still to be gained in developmental eye-tracking research. While it is increasingly more common to report eye-tracking data quality in adult eye-tracking research (Holmqvist et al., 2012), this is still rare in developmental eye-tracking research. Indeed, publishing guidelines from a prominent developmental journal do not require eye-tracking data quality metrics to be reported (Oakes, 2010).

### 1.3 The ugly, or, the present article

Given that eye-tracking data quality and analysis may not be of primary interest when an experimental study is conceived, it has perhaps been underexposed. A contributing factor may be that eye-tracking data quality is dependent on many different factors. Nyström et al. (2013), for example, have shown that the precision and accuracy of eye-tracking data depends on the skills of the operator, the calibration method, the eye color of the participant, and the time since the last calibration. In other words, eye-tracking data quality may be high or low for reasons related to the technical workings of the eye tracker, reasons related to participant physiology, or as a result of the choices made by an operator or researcher. The broader questions addressed in the present article can thus be stated as follows. First, how can data quality in developmental eye-tracking research be improved? Second, how can eye-tracking data of variable quality best be analyzed? The overarching issue is how to avoid that eye-tracking metrics are biased by data quality, so that individual developmental trajectories can be uncovered regardless of the eye-tracking data quality of an individual recording. In order to answer this question, we adopt the data-quality perspective and survey the eye-tracking data collection and analysis procedures in the YOUth study.

We use examples and eye-tracking data from the YOUth study, and focus specifically on the following three questions:

1. How can an eye-tracking setup be designed to optimize eye-tracking data quality in developmental eye-tracking research?
2. How can eye-tracking data quality be optimized when the researcher is not the primary person carrying out the recordings, but many research assistants fulfill this function?
3. What ranges of eye-tracking data quality are to be expected in developmental eye-tracking research, and how can eye-tracking data analysis be matched to the data-quality range?

We thus tackle the *ugly* problems that help ensure that valid conclusions about child development can be drawn. We end with advice for longitudinal and cohort-based eye-tracking research and generalizations to other methodologies (e.g. EEG).

## 2 Methods

In this study, we adopt the data-quality perspective in order to answer three questions related to optimizing eye-tracking data quality and analyzing eye-tracking data of potentially low quality. We first describe the eye-tracking data sets from the YOUth study used for this paper. Hereafter, we provide the necessary methodological background for each of our three questions.

### 2.1 Participants

The YOUth study is a large cohort study involving two cohorts (0–6 years and 9–15 years) with a projected 3000 participants in each. At each visit, multiple measurements are conducted, among which eye tracking, EEG, questionnaires, behavioral tasks, observation of parent-child interaction, biological material, and fMRI. The exact measurements conducted at each time point vary with age. Recruitment for the YOUth study commenced in 2015 and is still ongoing. Participants are recruited in Utrecht and its neighboring communities. The YOUth study was approved by the Medical Research Ethics Committee of the University Medical Center Utrecht and all participants’ parents provided written informed consent. A brief overview of the YOUth study including the measurements conducted at each timepoint is available from https://www.uu.nl/en/research/youth-cohort-study. Detailed information on the YOUth study design, in- and exclusion criteria, and the measurements conducted at each timepoint is forthcoming in this special issue.

For the present paper, 500 eye-tracking data sets were requested from the YOUth study for each available age group (5 months, 10 months, 3 years, 9 years). As the 3-year wave has only recently commenced, only 31 sets were available at the time of writing. Descriptive statistics of the eye-tracking data sets used are given in Table 1.

**Table 1:** Descriptive statistics of the eye-tracking data sets used for this article. RA = Research Assistant.

Data set	Number of participants	Age (years)	Sex (% female)
5 months – RA 1	119	0.46 (*sd* = 0.06)	57
5 months – RA 2	104	0.45 (*sd* = 0.06)	53
5 months – RA 3	48	0.45 (*sd* = 0.06)	50
5 months – RA 4	42	0.45 (*sd* = 0.07)	60
5 months – RA 5	38	0.46 (*sd* = 0.07)	39
10 months – RA 2	97	0.87 (*sd* = 0.07)	49
10 months – RA 1	92	0.88 (*sd* = 0.08)	48
10 months – RA 6	44	0.86 (*sd* = 0.07)	48
10 months – RA 7	35	0.89 (*sd* = 0.07)	40
10 months – RA 8	33	0.86 (*sd* = 0.06)	48
5 months	500	0.46 (*sd* = 0.06)	50
10 months	500	0.87 (*sd* = 0.07)	52
3 years	31	2.40 (*sd* = 0.27)^a^	58
9 years	500	9.50 (*sd* = 0.88)	54

a Note that our age groups are jittered around the target age. We have only had a few participants in the 3-year-old group as of this writing, which explains the low age (2.4 years) compared with the target age (3 years).

### 2.2 Apparatus and stimuli

Eye-tracking data were collected using the Tobii TX300 running at 300 Hz. Communication with the Tobii TX300 was achieved using the Tobii SDK controlled through MATLAB running on Mac OSX. PsychToolbox (Brainard, 1997) was used for stimulus presentation.

During the participant visits at 5 and 10 months, three eye-tracking experiments are conducted: a gaze cueing experiment, a gap-overlap experiment, and a free-viewing experiment. During the visits at 3 years, the three experiments are supplemented by a word-learning experiment. At the 9-year visit, the free-viewing experiment is replaced with an anti-saccade experiment. As we wanted to analyse eye-tracking data from the same experiment for each age group, we selected the gap-overlap experiment. The gap-overlap experiment is planned as the second of three (four for the 3-year-olds) experiments during the visit of the participant. As eye-tracking data quality tends to decrease as a function of time during the experiment (e.g. Nyström et al., 2013, Hessels et al., 2015a), data quality measures from this experiment are expected to be most representative of eye-tracking data quality in our entire set of eye-tracking experiments.

In the gap-overlap experiment, a central stimulus is presented followed by a peripheral one at either side of the screen. By varying whether the central stimulus disappears prior to peripheral stimulus onset or not, attentional disengagement is investigated (see e.g. Saslow, 1967, Elsabbagh et al., 2013, Van der Stigchel et al., 2017). Prior to the experiment, a 5-point operator-controlled calibration procedure was conducted. Each calibration point consists of a rotating colored spiral that contracts when the operator presses a button. The calibration output of the Tobii SDK was inspected, and, when necessary, individual points could be recalibrated. After calibration was deemed to have been successful, or when the participant was deemed to be losing attention, the experiment commenced. Detailed information on the calibration procedure is given in Hessels et al. (2015a).

### 2.3 Operationalizations of eye-tracking data quality

As noted before, eye-tracking data quality is usually characterized by three aspects: precision (or variable error), accuracy (or systematic error) and data loss. In the present article, we only present measures of precision and data loss, not accuracy. Precision and data loss can directly affect the processing of the gaze-position signals. Large variable errors (low precision) can be, for example, problematic for fixation classification (Wass et al., 2014, Holmqvist et al., 2012, Hessels et al., 2017). Large proportions of data loss may mean that the gaze-position signal is not analyzable at all, while small periods of data loss can also be problematic for fixation or saccade classification (Wass et al., 2014, Hessels et al., 2017). Accuracy (or systematic error) is not evident from the gaze-position signal alone. It requires a known (or assumed) fixation location to which the gaze-position signal is compared. In adult research, assuming such a location is straightforward: adults can be instructed to look somewhere. In infant research, this is not as trivial. Furthermore, systematic errors do not directly affect e.g. fixation or saccade classification in the gaze-position signal. The interested reader is referred to other psychology literature. Estimates of systematic errors in infancy, toddlerhood, school age and adults are given in Hessels et al. (2015a) and Dalrymple et al. (2018). Frank et al. (2012) furthermore describe a method of re-calibrating eye-tracking data to remove large systematic errors and Orquin and Holmqvist (2018) describe how Area of Interest (AOI) size may be chosen such that it can accommodate systematic errors of known size.

Precision and data loss were operationalized as follows. For precision, a 100 ms window was slid over the horizontal and vertical gaze-position signals of each eye for each trial. For each window, the Root Mean Square sample-to-sample deviation (RMS s2s) was calculated. The median of all computed RMS s2s values in a trial was then determined, and these medians were averaged over trials. The horizontal and vertical components were combined using Pythagoras’ theorem to acquire an RMS s2s value for the gaze-position signal of each eye. These two values were subsequently averaged to acquire the final estimate of precision of the eye-tracking data acquired during each participant's recording. For data loss, the proportion of samples in a trial without a reported gaze coordinate was determined for each eye. These values were averaged over trials and subsequently averaged over eyes to acquire the final estimate of data loss for the eye-tracking data of each participant. Lower values for the RMS-s2s deviation and the proportion of data loss indicate better eye-tracking data quality.

### 2.4 Question 1 – Defining the requirements for an eye-tracking setup

The first question we aim to answer is how to design an eye-tracking setup in order to optimize eye-tracking data quality in developmental eye-tracking research. With an eye-tracking setup, we mean the combination of an eye tracker, a computer screen, a seat for the participant, and whatever table or mounting device is needed to position the eye tracker. This setup should allow the eye tracker and participant to be positioned and oriented optimally with respect to one another. Optimal here means the relative position and orientation between eye tracker and participant for which the quality of recorded eye-tracking data is best, while the participant is still able to conduct the task. Often, the optimal position and orientation are specified by the eye-tracker manufacturer by means of a tracking distance between the eyes and eye tracker, and a so-called head box. This head box is a theoretical box (or more appropriately, a *viewing frustum*) in front of the eye tracker in which the participant should be able to move while a gaze position can still be reported by the eye tracker. In adult eye-tracking research, head movement may be restrained through the use of a chin rest or bite bar (e.g. Engel, 1977, Collewijn and Tamminga, 1984), thereby ensuring that the optimal position and orientation are maintained throughout a recording. In developmental research, however, participants tend to move, which can be problematic for eye-tracking data quality. Wass et al. (2014), for example, showed that when head movement occurs, the variable error in the eye-tracking data and data loss increase (although see Hessels et al. (2015a) for potential problems of using the eye-tracker signal to estimate head movement). It has further been shown that some eye trackers are more susceptible to participant movement than others: some eye trackers do not report a gaze position anymore when a participant tilts their head slightly from the optimal position and orientation, whereas others do (Hessels et al., 2015b, Niehorster et al., 2018). Even for eye trackers that do still report a gaze position, eye-tracking data quality decreases during participant movement and under non-optimal head orientations with respect to the eye tracker. An important question for developmental eye-tracking research thus is: how can children be positioned such that participant movement is reduced and eye-tracking data quality does not decrease and the participant can still complete the task?

While older children can be positioned using regular office chairs and chin rests, this is not possible with toddlers and infants. Seating practices with young children differ between developmental eye-tracking labs. For example, some researchers position infants in the parent's lap (Gredebäck et al., 2010, Wass and Smith, 2014), whereas others position infants in a car seat (Shic et al., 2014, Jones et al., 2008). The different types of seating afford different amounts of movement, yet also put different constraints on the rest of the geometry. For example, in a car seat, the infant is strapped in, which means there is typically little movement except perhaps head sway. However, an infant laying in a car seat is not oriented optimally relative to an eye tracker positioned on a table. An easy rule of thumb is that when the eye tracker is mounted underneath a screen, that screen should be positioned parallel to the participant's head. The eye tracker will then be directed towards the eyes from below the participant's line of sight when that participant is looking straight ahead. With an infant in a car seat, however, the eye tracker likely needs to be mounted on an arm of some sorts to allow it to be positioned in the optimal orientation with respect to the infant. When an infant is seated in the parent's lap or in a high chair, positioning the infant's head parallel to a computer screen on a table, with the eye tracker underneath, is easier. Yet, these situations afford more movement than a car seat. In the only direct comparison to date, the best accuracy of the gaze position signal was achieved in recordings with infants in a car seat, rather than recordings with infants in a high chair or directly on the parents’ lap (Hessels et al., 2015a, although the comparison was not systematic, i.e. the car seat was the default choice). Restricting infant movement is thus possible through the use of a car seat, and while it may increase eye-tracking data quality, it constrains how the eye tracker can be positioned relative to the participant.

In the YOUth cohort studies, we measure eye movements from children as young as 5 months up to children aged 15 years. As we wanted to keep the eye-tracking setup as similar as possible across ages, the setup needed to satisfy the following criteria:

1. The eye tracker should be able to measure eye movements from infants, toddlers, school-aged children and adults.
2. The eye tracker should be robust to participant movement, with which we mean that a gaze position can still be reported by the eye tracker when the participant has moved.
3. The setup should afford as little movement of the participant as possible.
4. The participant can be positioned parallel to a computer screen, with the eye tracker mounted underneath in the optimal relative orientation to the participant.
5. The setup should be adaptable to different age-groups within one cohort (0–6 years, 9–15 years).

### 2.5 Question 2 – Research assistants as the operators in eye-tracking research

In many longitudinal or cohort-based studies, as in the YOUth study, most recordings are conducted by research assistants (RAs). Obviously, these RAs need to be trained in the eye-tracking recording procedure. It is of particular importance to train RAs well, as eye-tracking data quality is known to be affected by operator expertise (Nyström et al., 2013). The question is how to organize the training protocol so that RAs are trained effectively and efficiently.

Although it is outside the scope of the present article to go into the full training protocols in the YOUth cohort studies, we briefly outline the protocol for the eye-tracking domain. RAs begin by reading the protocols for the eye-tracking data collection, which are specified for each experiment. Second, RAs are expected to watch a short lecture on the basics of eye tracking, and a number of videos on positioning and calibration. Third, RAs have to observe at least 1 recording from a more experienced RA. Fourth, the RA participates in a training session conducted by the head of the eye-tracking domain (author RH) or a delegated party. This training session focuses on positioning, calibration and eye-tracking data monitoring during the experiments. Fifth, RAs observe two more recording from a more experienced RA. Sixth, RAs conduct at least three recordings while being observed by a more experienced RA. Hereafter, RAs are qualified to conduct recordings by themselves. The head of domain or a delegated party conducts checks of the new RAs and random checks every few months.

An important question is whether the different RAs are all able to obtain high quality eye-tracking data during the recordings in which they serve as the operator. While we did not conduct any systematic investigations of whether our training protocol is effective, for obvious reasons of project continuity, we will investigate whether eye-tracking data quality measures differ substantially between RAs. We therefore compared estimates for precision and data loss for the five RAs that conducted the most recordings with 5-month-old and 10-month-old infants (see Table 1). Note that two RAs were among the top 5 RAs that conducted the most measurements for both the 5-month and 10-month age groups (RAs 1 & 2)

### 2.6 Question 3 – What ranges of eye-tracking data quality to expect

The third question that we address in this article is what ranges of eye-tracking data quality are to be expected in developmental eye-tracking research, and how eye-tracking data of potentially low quality can be analyzed. Despite all efforts to improve eye-tracking data quality through optimized setups and substantial training protocols, it is highly unlikely that eye-tracking data for infants, toddlers and school-aged children will be of equal quality. It is thus imperative to consider the ranges of eye-tracking data quality that are typically obtained across ages, and how this affects eye-tracking data analysis. We therefore analyzed data quality ranges for precision and data loss for the age groups that are already in progress in the YOUth study: 5 months, 10 months, 3 years (recently started) and 9 years. We then discuss constraints on data-analysis tools, and discuss age-specific data-analysis problems. Eye-tracking data quality measures are reported for 500 participants per age group, except for the 3-year-old age group, for which only 31 participants had been recorded at the time of writing. As before, estimates for precision and data loss are presented (see *Operationalizations of eye-tracking data quality*), both derived from the gap-overlap experiment. Descriptive statistics of the eye-tracking data sets per age group are given in Table 1.

## 3 Results

### 3.1 The eye-tracking setup in the YOUth study

**Fig. 2:**
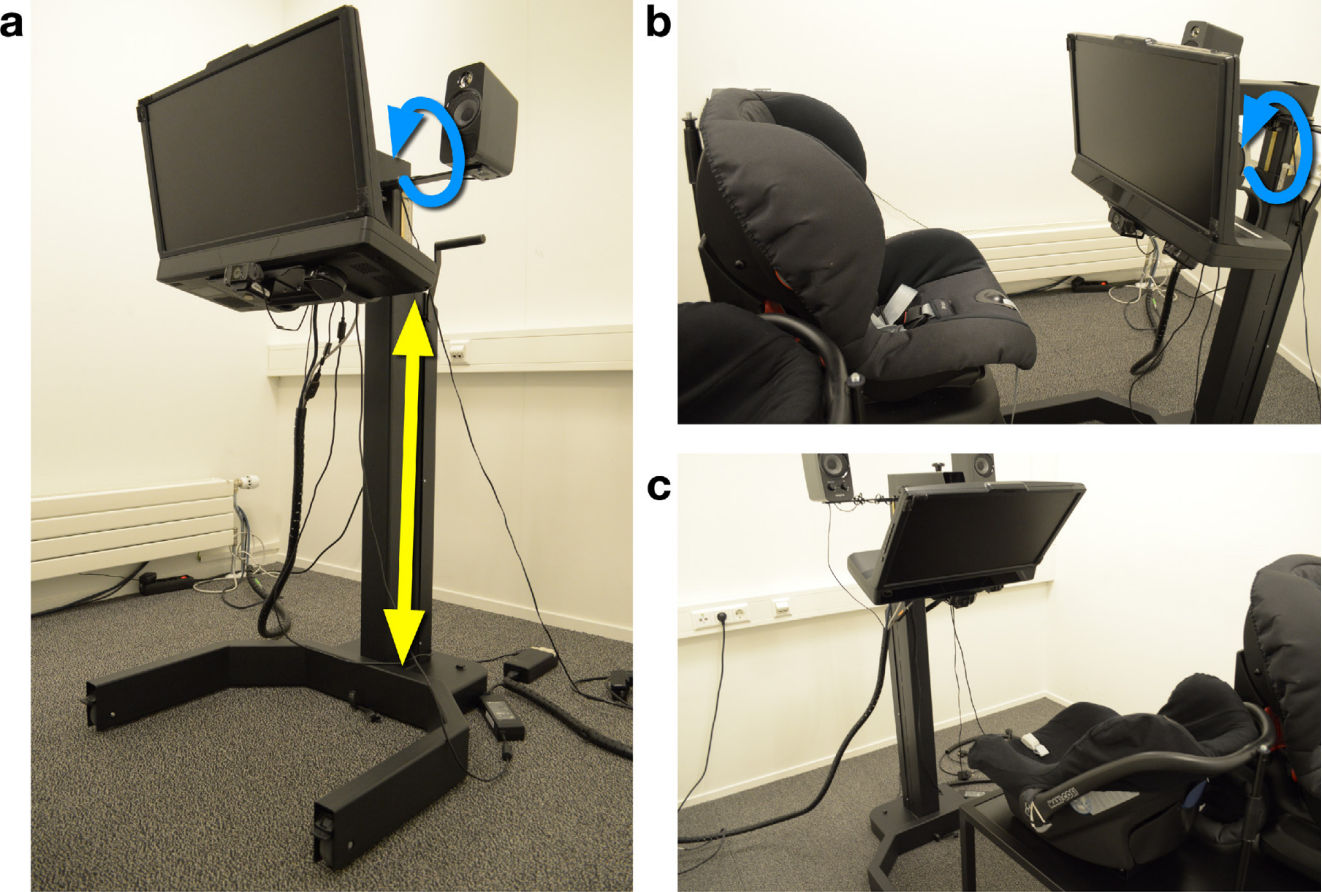
(a) Eye-tracking setup used in the YOUth cohort study for ages 0 and up. The eye tracker (Tobii TX300) is mounted in a frame that can be lowered and heightened (indicated by the yellow arrow). The eye tracker can be tilted from fully vertical to fully horizontal (indicated by the blue arrow), such that the optimal relative position and orientation between eye tracker and participant can be achieved with almost all manners of seating. (b) The setup as it is generally positioned with larger infants and toddlers. (c) The setup as it is generally positioned with young infants. The seats in (b) and (c) are mounted on a platform with wheels. (For interpretation of the references to color in this figure legend, the reader is referred to the web version of this article.)

Fig. 2 depicts the setup developed for the YOUth study. It is a custom design, built by a professional constructor according to the needs specified by the eye-tracking researchers in the YOUth cohort study. It consists of an eye tracker mounted to a moveable frame and a platform with two different seats mounted on it. The eye tracker we chose to use in the YOUth study is the Tobii TX300. This decision was based on extensive testing of different eye trackers on their robustness to participant movement and non-optimal head orientation relative to the eye tracker (Hessels et al., 2015b, Niehorster et al., 2018). The eye tracker is mounted underneath a computer screen. As such, the optimal position and orientation between participant and eye tracker can be achieved by (1) orienting the computer screen parallel to the participant's head, and (2) positioning the computer screen at the right distance and height relative to the participant's line of sight when looking straight ahead.

As denoted by the yellow arrow in Panel A in Fig. 2, the computer screen and eye tracker can be lowered and heightened. As denoted by the blue arrow, it can furthermore be tilted from fully vertical to fully horizontal relative to the floor. It is thus possible to position and orient the eye tracker optimally with respect to the participant with many different types of seating,^3^ for example with a small infant car seat (Panel C) or a slightly larger car seat (Panel B). It is also possible to position the eye tracker fully upright and use it with older children or adult participants positioned in a chin rest. This setup makes it possible to conduct eye-tracking research with participants from infancy to adulthood. In order to facilitate quick positioning of the children, two car seats are mounted on a wheeled platform: one seat for infants, one for larger infants or toddlers. Both seats afford little participant movement. The wheeled platform makes it easy to fine tune the exact positioning of the child, without having to ask a parent to move, or to move the eye-tracker and its frame. In sum, the presented setup satisfies all criteria we noted in the *Methods* section.

### 3.2 Eye-tracking data quality for multiple research assistants

**Fig. 3:**
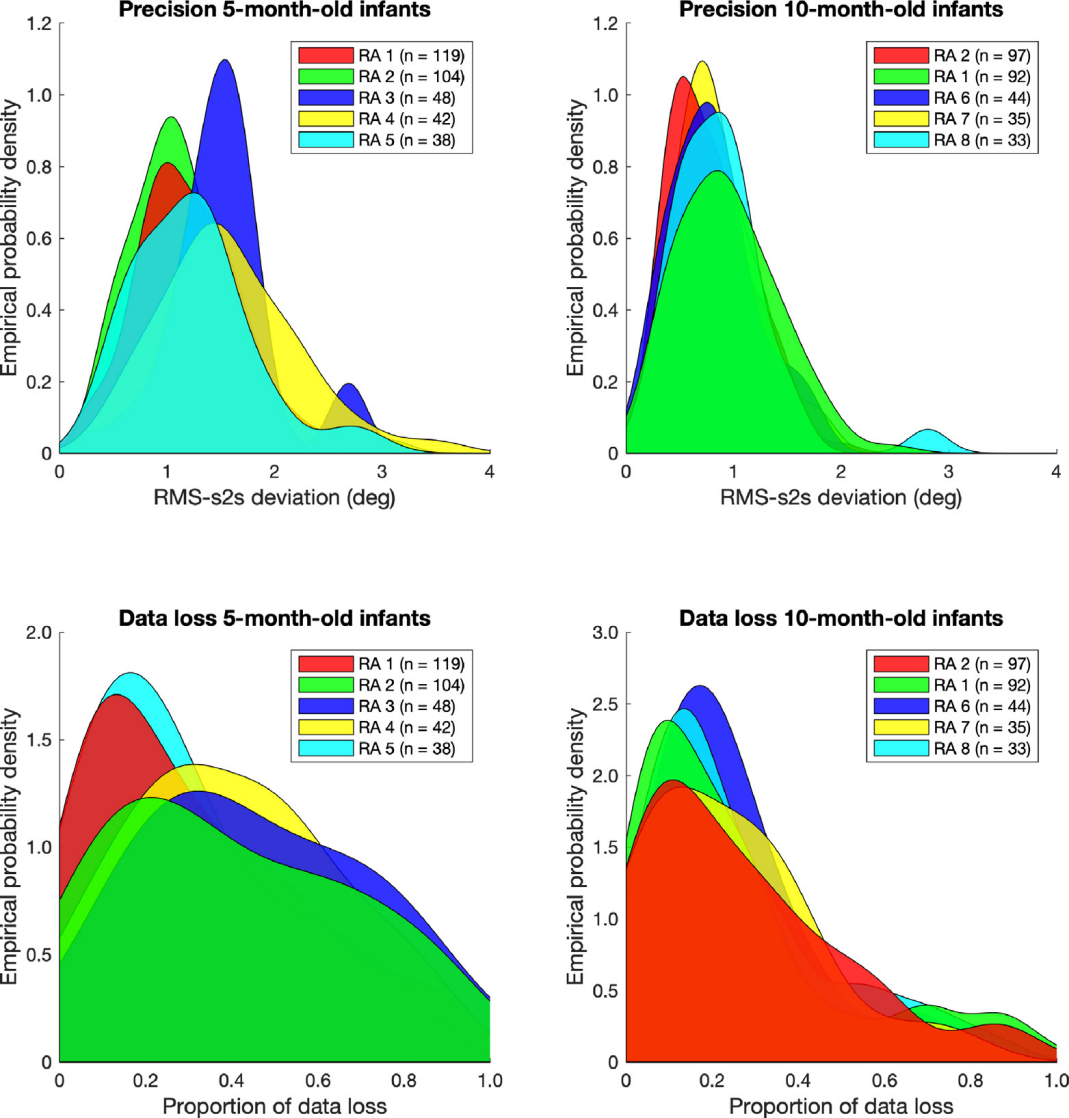
Distributions of the estimates for precision for 5-month-old infants (top left) and 10-month-old infants (top right) and data loss for 5-month-old infants (bottom left) and 10-month-old infants (bottom right). Precision was estimated by the Root Mean Squared sample-to-sample deviation (RMS s2s) of the gaze-position signals. Data loss was estimated as the proportion of samples without a gaze coordinate. Each distribution belongs to one research assistant (RA) and is kernel-smoothed to ease visual comparison. The number of recordings for each RA is given in parentheses. The values on the *y*-axes are empirical probability densities for the smoothed empirical distribution. The area under the curve of each smoothed empirical distribution is 1.

Fig. 3 depicts distributions of the estimates for precision and data loss for the 5-month-old and 10-month-old infants, as recorded by the 5 RAs that conducted the most recordings per age group. Each participant contributes one value to a distribution. In order to ease visual comparison of the distributions, they were kernel-smoothed in MATLAB.^4^ As visible from the top left panel in Fig. 3, the distribution of precision for 5-month-old infants was not identical for every RA. For RAs 3 and 4, for example, the peak of the distribution is close to 1.5° of RMS-s2s deviation, while it peaks around 1° for RAs 1 and 2. This is not the case for 10-month-old infants as seen from the top right panel, at least not to the same extent. Here, the distributions peak roughly at the same value for all RAs. As the distributions overlap for a large part, statistical analysis was conducted to support these findings. A one-way Bayesian ANOVA was conducted in JASP (JASP Team, 2018) for the RMS s2s values with RA as a fixed factor. This was done separately for the 5-month-old and 10-month-old infants. For the 5-month-old infants, the model including the RA factor was best supported by the data, as indicated by a Bayes Factor (BF_*M*_, where _*M*_ denotes the model described) of 6.5 * 10^4^. This indicates that the RA affects the RMS s2s value. For the 10-month-old infants, the null model was supported best by the data, albeit only slightly (BF_*M*_ = 2.66).

For data loss, a similar pattern is observed. As visible from the bottom left panel in Fig. 3, the distributions of data loss for 5-month-old infants have a sharp peak at around 0.15 for RAs 1 and 5, while the distributions are much wider for RAs 2, 3 and 4. For the 10-month-old infants (bottom right panel), the distributions of data loss of the RAs peak closer together. These findings were again supported by statistical analysis. One-way Bayesian ANOVAs were conducted for the proportion of data loss values with RA as a fixed factor, separately for the 5-month-old and 10-month-old infants. For the 5-month-old infants, the model including the RA factor was best supported by the data (BF_*M*_ = 6.06). For the 10-month-old infants, the null model was supported best by the data (BF_*M*_ = 75.01).

Together, these findings indicate that eye-tracking data quality may be dependent on the RA. Here, eye-tracking data quality was RA-dependent for the younger infants (5 months) but not for the older infants (10 months). It should be noted, however, that the RAs were not the same for the 5-month-old and 10-month-old infants. The reason for this is that only two RAs that conducted more than 30 measurements for the 5-month-old infants also conducted more than 30 measurements for the 10-month-old infants. When fewer than 30 measurements are available, estimates of precision and data loss for an RA are unreliable, as they can be too dependent on a few ‘easy-’ or ‘difficult-to-record’ infants. However, in order to check that both age and RA experience contribute to eye-tracking data quality, and differences between age groups are not due to different RAs, a Bayesian ANOVA was conducted for the two RAs that contributed measurements to both age groups. For the estimates of precision, this revealed that the model including both the participant age group and the RA as a factor was best supported by the data (BF_*M*_ = 22.26). For the estimates of data loss, the model including only participant age group was best supported by the data (BF_*M*_ = 3.37), followed closely by the model including both participant age group and RA (BF_*M*_ = 2.48). Eye-tracking data quality is thus dependent on both participant age and which RA conducts the measurement.

Another interesting finding is that it is not necessarily the case that more experienced RAs, i.e. those with more recordings to their name, are the ones with the best eye-tracking data quality for their recordings. The ‘best’ distribution for both measures of eye-tracking data quality is one that peaks early and does not have a long tail to the right. For the precision observed with 5-month-old infants, RA 2 fits this description best, followed by RA 1 and RA 5, showing that more experienced operators produced better eye-tracking data quality. However, for the data loss observed with 5-month-old infants, RA1 and RA 5, not RA 2, fit this description best. For the 10-month-old infants, this comparison is more difficult to make, as the distributions are more alike.

### 3.3 Eye-tracking data quality ranges in developmental eye-tracking research

**Fig. 4:**
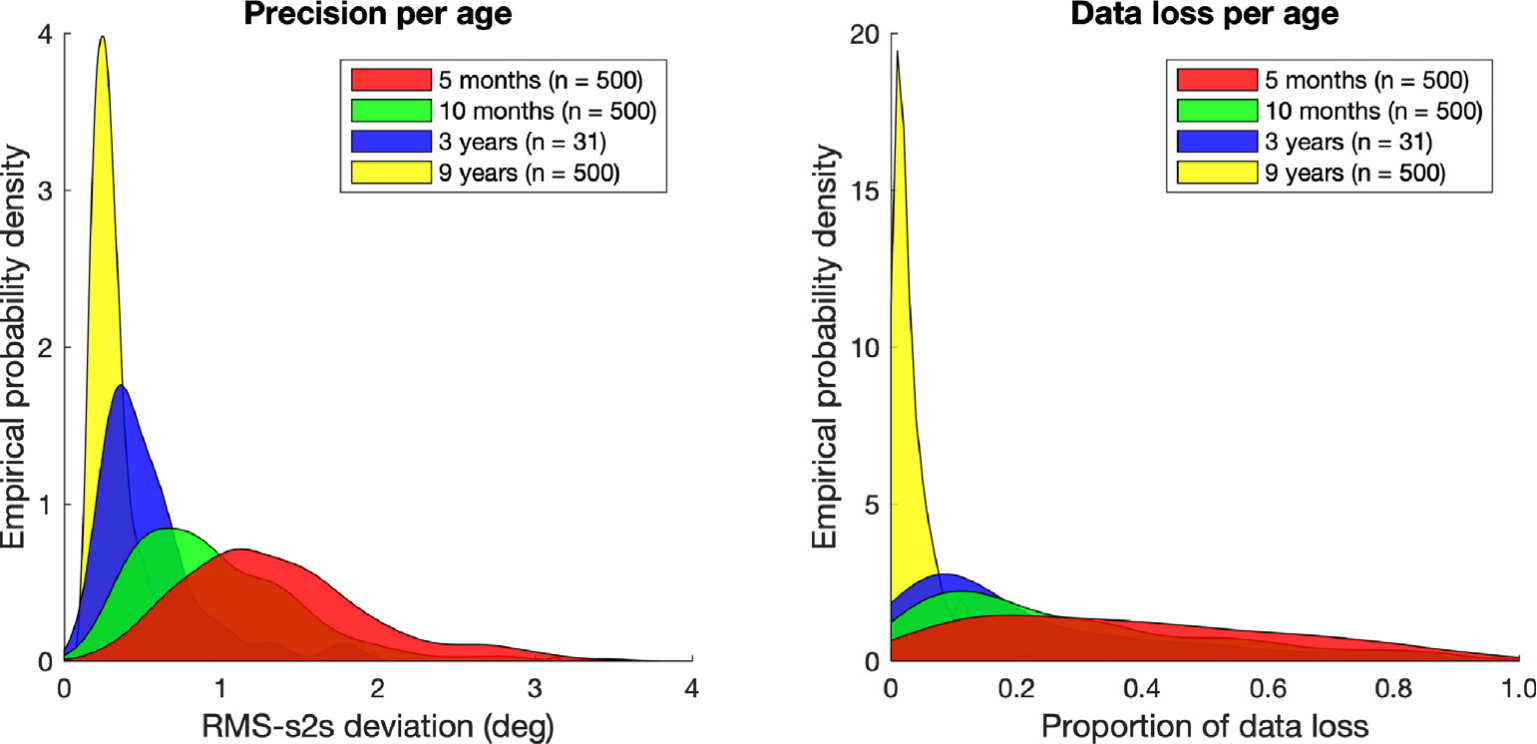
Distributions of the estimates for precision (left) and data loss (right) for the four different age groups: 5 months, 10 months, 3 years, and 9 years. Precision was estimated by the Root Mean Squared sample-to-sample deviation (RMS s2s) of the gaze-position signals. Data loss was estimated as the proportion of samples without a gaze coordinate. Distributions are kernel-smoothed to ease visual comparison. The number of recordings for each age group is given in parentheses. The values on the *y*-axes are empirical probability densities for the smoothed empirical distribution. The area under the curve of each smoothed empirical distribution is 1.

Fig. 4 depicts distributions of the RMS-s2s deviation (estimate for precision) and proportion of data loss for the four age groups. Each participant contributes one value to a distribution. As is visible from the left panel, the distribution of RMS-s2s deviation peaks around 1 to 1.5° for the 5-month-old infants, while it peaks increasingly earlier for the 10-month-old infants, the 3-year-old children and the 9-year-old children. A similar pattern is observed for the proportion of data loss (right panel in Fig. 4). Here, the distribution for the 5-month-old infants is very wide, with no clear peak. For the older ages (10 months, 3 years and 9 years), the distribution is increasingly more peaked towards the lower values, indicating better eye-tracking data quality. Note that for both RMS-s2s deviation and the proportion of data loss, the distributions are wider for younger children than for older children. This means that the differences in data quality are larger between the eye-tracking data of the younger participants, than the eye-tracking data of the older participants.

**Fig. 5:**
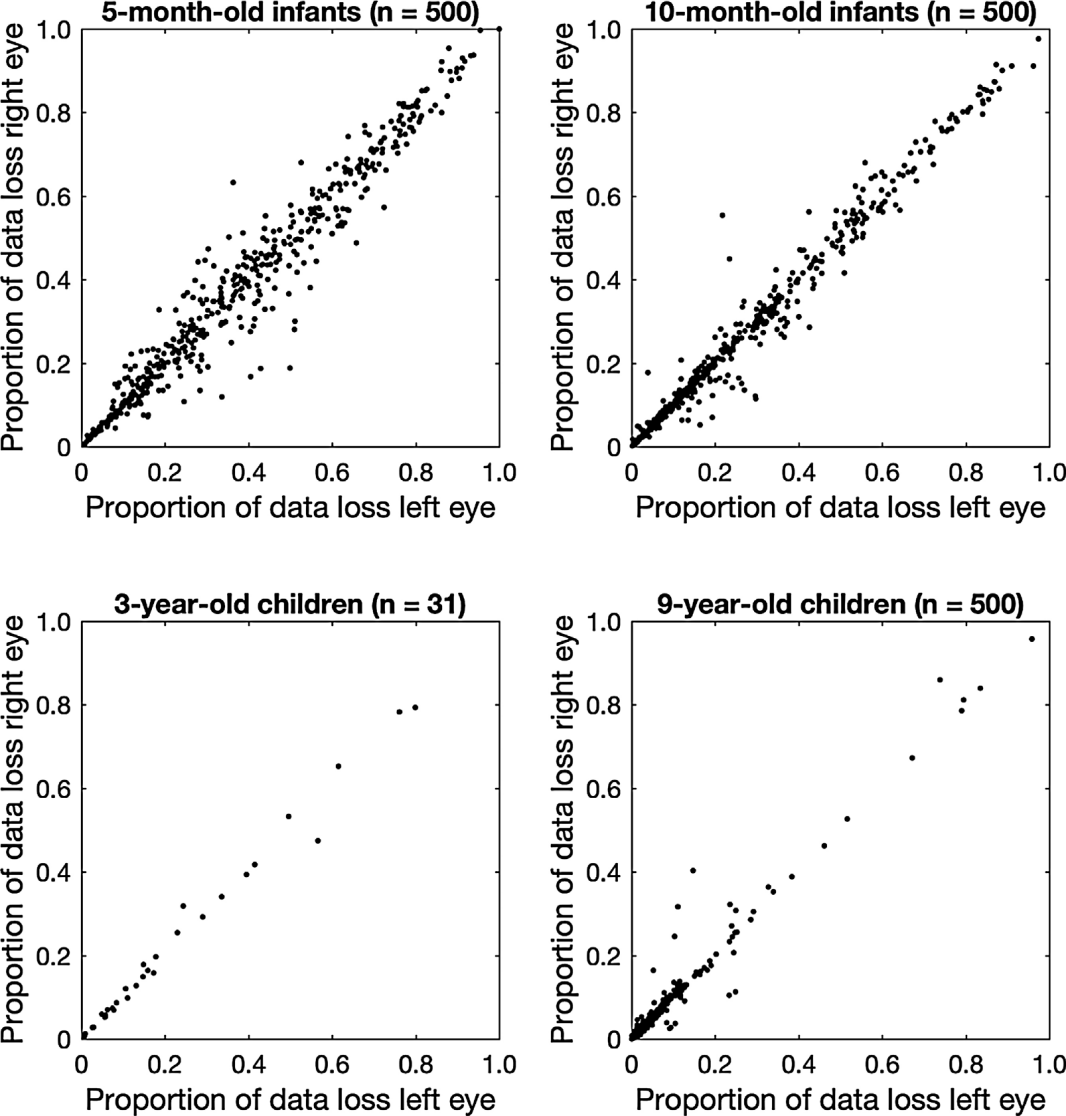
Proportion of data loss for the left eye plotted against the proportion of data loss for the right eye for the four different age groups: 5 months (top left), 10 months (top right), 3 years (bottom left), and 9 years (bottom right).

We further investigated differences in data loss across ages, by looking at data loss for the individual eyes. Ideally, data loss is 0 for both eyes, indicating that a gaze position could always be reported for each eye. However, data loss is inevitable and particularly so in developmental eye-tracking research. If a participant blinks, for example, a gaze coordinate cannot be reported for a short period (generally a few hundred milliseconds at maximum). If a participant looks away from the eye tracker completely, a gaze coordinate can also not be reported. Such episodes are likely to occur with infants or toddlers. However, data loss can also occur when a participant is not blinking or looking away. As stated, such data loss may occur due to technical difficulties in tracking the eyes (Wass et al., 2014, Hessels et al., 2015a), for example, when a pupil cannot be found in the camera image of the eye tracker. When a participant blinks or looks away, data loss occurs for both eyes at the same time. If all data loss in a given experiment is due to blinks or looking away, one would thus expect the proportion of data loss to be nearly identical for the two eyes. This does not need to be the case when data loss occurs for different reasons. We therefore plotted the proportion of data loss for the left eye against the proportion of data loss for the right eye in Fig. 5.

As visible from the top left panel in Fig. 5, the proportion of data loss for 5-month-old infants ranges from 0 to 1. Interestingly, there is quite some spread around the unity line, which indicates that for these participants, one eye had a consistently higher proportion of data loss than the other. As visible from the top right panel, the proportion of data loss for 10-month-old infants also ranges from 0 to 1, but the spread around the unity line is substantially smaller. For the 3-year-old and 9-year-old children (bottom two panels), the spread around the unity line further decreases, and the proportion of data loss clusters more in the bottom left corner of the plot. These findings can be described by two metaphorical forces. As children age, the proportion of data loss is forced towards the unity line, and towards 0 (the bottom left corner of the plot). In other words, as children age the proportion of time that only one eye can be tracked is reduced and the amount of data loss that occurs at all. This furthermore indicates that the higher levels of data loss for the 5-month-old infants compared with the 10-month-old infants are not likely due to more looking away from the screen, which would lead to both eyes not being tracked instead of only one.

## 4 Discussion

High data quality is the foundation on which solid developmental eye-tracking research is built. However, eye-tracking data quality has been underexposed in the developmental literature. In this article, we have questioned how knowledge on eye-tracking data quality could be used to improve eye-tracking recordings and analyses in longitudinal research so that valid conclusions about child development may be drawn. In order to answer this broader question we adopted a data-quality perspective and aimed to answer the following three research questions:

1. How can an eye-tracking setup be designed to optimize eye-tracking data quality in developmental eye-tracking research?
2. How can eye-tracking data quality be optimized when the researcher is not the primary person carrying out the recordings, but many research assistants fulfill this function?
3. What ranges of eye-tracking data quality are to be expected in developmental eye-tracking research, and how can eye-tracking data analysis be matched to the data-quality range?

Regarding the first question, we have shown how our eye-tracking setup was designed to optimize the quality of the eye-tracking data recorded. It was designed to facilitate obtaining the optimal relative position and orientation of the eye tracker and the participant, for infants, toddlers, school-aged children and adults. Regarding the second question, we have outlined our RA-training protocol and have shown that eye-tracking data quality can be RA-dependent even after a thorough training protocol. Regarding the third question, we have reported distributions of precision and data loss measures for four age groups (5 months, 10 months, 3 years, and 9 years), based on 1531 recordings. We will discuss below how differences in these distributions of eye-tracking data quality measures constrain data analysis in longitudinal and cohort-based eye-tracking research.

The fact that eye-tracking data quality is RA-dependent is an important factor to consider when monitoring eye-tracking data quality in large studies. Importantly, the magnitude of differences in eye-tracking data quality achieved by different RAs, could approximate the difference in eye-tracking data quality between age groups (5-month-olds and 10-month-olds). This highlights that even a thorough training protocol with many checks by experienced RAs and researchers does not necessarily mean that eye-tracking data quality will be RA-independent. Yet, although our findings indicate that eye-tracking data quality is RA-dependent, and is not necessarily highest for more experienced RAs, they should not be interpreted without considering other aspects of the recording. It may be the case, for example, that the different RAs also have different thresholds for when to start, stop or pause an ongoing recording: an important aspect of the recording that we do not explicitly consider here. However, our findings do indicate that it is worthwhile to monitor RA-dependence of data quality, and potentially focus training efforts on those RAs with consistently lower eye-tracking data-quality values. An important outstanding question is exactly what drives the differences in eye-tracking data quality between the different RAs. One problem is that errors early in a measurement may propagate. For example, problematic positioning of an infant may lead to more calibrations needing to be conducted, potentially leading to an infant that becomes fussy before the experiment is over. In the experience of the first author (RH) in supervising the RAs, the most difficult decision is when to start the experiment and when to keep tweaking and fine-tuning the positioning and calibration.

The distributions of eye-tracking data quality, specifically precision and data loss, that we have reported confirm previous work on the differences in precision between age groups (Dalrymple et al., 2018, Hessels et al., 2016b, Hooge et al., 2018b), and extend it by showing how data loss differs across age groups. Furthermore, our distributions for precision and data loss were acquired with a large number of participants per age group: 500 children for all age groups except the 3-year-olds. While eye-tracking data quality measures may differ as a result of the specific eye tracker used, the demographics of the participant groups or the lab at which the research is conducted, we hypothesize that the relative differences between age groups will generalize beyond our eye tracker and participant demographics. Finally, we have highlighted important differences in the nature of data loss between the age groups. The question then beckons: how do these findings constrain eye-tracking data analysis and the conclusions that may be drawn from it?

We first address the differences in precision across age. Our analyses of the precision poses two specific problems for developmental eye-tracking data analysis: (1) precision is increasingly better for the older ages, and (2) the differences within an age group are increasingly smaller for the older ages. As stated before, a low precision (or higher variable error) may affect the number of fixations and corresponding fixation durations classified in eye-tracking data (Wass et al., 2014, Holmqvist et al., 2012, Hessels et al., 2017). Should one thus use a fixation-classification algorithm for which the output is *not* precision-independent, one might conclude that (1) the number of fixations or average fixation duration changes as a function of age, and (2) individual differences in these measures become smaller with age, *without* there being any actual difference in the underlying gaze behavior. These conclusions may purely depend on the precision of the eye-tracking data recorded. Clearly, this is an undesirable situation. Hessels et al. (2017) have recently developed a fixation-classification algorithm which is less susceptible to differences in precision between 0 and 2° of RMS-s2s deviation than competing algorithms. While this is an important step forward in analysis of low-quality eye-tracking data, there can still be a substantial proportion of participants in infant research for which the average RMS-s2s deviation is larger than 2° (see Fig. 4).

With regard to data loss, the problem is likely more complex. If the proportion of data loss is close to 1, it is unlikely that the remaining gaze data is going to be useful when drawing conclusions about a child's gaze behavior. However, large proportions of data loss need not be problematic. If, for example, an infant looks away from the screen and eye tracker for 50 to 60% of the time, it may still be that the eye-tracking data for the remaining time is perfectly analyzable. In that scenario, time spent looking away from the screen and eye tracker may be an informative measure in itself, for example of interest in the visual stimulus. Yet, when periods of data loss are very short (<100 ms) and occur often, this can be problematic for fixation or saccade classification (Wass et al., 2014, Hessels et al., 2017). Moreover, if data loss occurs for one eye, but not the other, the question then beckons how gaze-position signals of the two eyes (if both recorded) should be analyzed. If one wants to average the gaze-position signals from the two eyes to acquire a lower precision due to the square root law (Hooge et al., 2018a), the eye with the largest proportion of data loss determines the total proportion of data loss, i.e. the worst signal in terms of data loss dominates. If one wants to maximize the available gaze-position data, one might choose the eye with the lowest proportion of data loss, or take a gaze position from whatever eye is available at any point in time. The latter option may be tricky: if one averages the gaze positions of two eyes when available and takes the gaze position of one eye when the other could not be reported, large shifts in the gaze position may occur. The shift may be due to, for example, different systematic errors for each eye.

Finally, it is important to consider what may drive the differences in eye-tracking data quality between age groups and whether some of the causes can be mitigated. Our findings not only show that eye-tracking data quality improves with participant age, but also that the nature of at least one aspect of eye-tracking data quality (data loss) differs between age groups. For the 5-month-old infants, for example, the proportion of time that one eye, but not the other, could be tracked was higher than for the 10-month-old infants. Given that many things can affect eye-tracking data quality, such as positioning, movement, eye physiology, but also infant fussiness and crying, it is not possible to pinpoint one cause for developmental differences in data quality based on our data. In order to provide a more concrete answer to this question, one might have to videotape the participant during the eye-tracking recording, to get an idea of how movement or positioning over time might affect eye-tracking data quality. However, at least part of the answer is hidden in the black box of the commercial eye tracker: Most eye-tracking manufacturers do not specify how the image of the eye is processed in order to estimate gaze position. Open-source eye trackers may be helpful here, although they are often not specifically developed for developmental eye-tracking research.

Based on the potential problems discussed above, we advise developmental eye-tracking researchers to always verify whether their fixation- or saccade-classification algorithms (or other analysis tools) are suited for the quality of the eye-tracking data obtained. This can be done, for example, by inspecting the raw gaze-position signals overlaid with the classified fixations for these signals. Critically, the assessment of whether an algorithm is suitable or not depends at least on (1) the analysis tool used, (2) the quality of the eye-tracking data recorded, and (3) the eye-movement measure of interest.

### 4.1 Advice

Based on our experiences and findings, we advise (prospective) developmental eye-tracking researchers in longitudinal and cohort-based research:

1. To consider an eye-tracking setup as at least a combination of an eye tracker, seating and table. Different seating puts different constraints on the relative positioning and orientation of the eye tracker with respect to the participant (and vice versa). This is also an important consideration for budgets in grant applications.
2. To consider that the operator can affect the data quality of the eye-tracking recording. Eye-tracking data quality can be optimized by ensuring that operators are well trained. Sharing experiences among operators, or comparing an operator's subjective assessment of a measurement with the data quality obtained, may be helpful in this regard.
3. To consider that systematic monitoring of eye-tracking data quality can be useful in spotting areas for improvement, for example in the RA-training protocol.
4. To always verify that the analysis tools used are suited for the quality of the eye-tracking data. If so required, specialized fixation- or saccade-classification algorithms can be applied. For example, some fixation-classification algorithms do not produce a valid output when the precision is low. Comparing the fixation-classification output to the raw data delivered by the eye tracker (e.g. gaze position signals as a function of time) may be useful to establish whether this is the case (see e.g. Hessels et al., 2017, Figure 10).
5. To report measures for eye-tracking data quality (precision, accuracy and data loss), and link these measures to the analysis tools used. Investigating, for example, average fixation durations puts more constraints on data quality than an AOI-based analysis of total looking time, especially when AOIs are large and the stimulus is sparse (e.g. Hessels et al., 2016b). An example of this can be found in Van der Stigchel et al. (2017), who report measures for the precision and accuracy obtained and state that these are within the values required for their fixation-classification algorithm and AOI analysis. See also Holmqvist et al. (2012) for advice on reporting eye-tracking data quality.
6. To realize that data quality can be an important factor in developmental eye-tracking research. When comparing different groups (by age or e.g. diagnostic status), investigating individual differences, or mapping an individual developmental trajectory, it is essential to consider whether eye-tracking data quality can explain the observed differences. If, for example, measures of precision, accuracy, or data loss differ significantly between groups or timepoints, researchers might wish to verify that this did not affect the computation of the eye-tracking measures used (see points 4 & 5).

Finally, we hope that developmental journals will consider the advice above, and adopt a policy for reporting eye-tracking data quality measures. Such a policy could take the form of requiring authors (1) to provide empirical values for precision, accuracy and data loss, (2) to make explicit whether these values differed between groups, timepoints or individuals (when relevant) and (3) to make explicit whether the values obtained were within range for the analysis tools used.

Although this article concerns the collection, quality and analysis of eye-tracking data, many of the problems addressed generalize to other neurocognitive domains as well. The basic principles of optimizing data quality by fine-tuning the setup and data-collection protocols transfer to any measurement technique, be it eye tracking, EEG, fMRI, or behavioral experiments. The important question is how the factors that affect data quality can best be understood and mitigated so that valid conclusions about child development may be drawn. We hope the present article furthers the debate on this topic.

## Declarations of interest

None declared.

## Footnotes

1 see https://www.uu.nl/en/research/youth-cohort-study for more details.

2 A ‘fixation’ generally refers to a period in which gaze is directed at a constrained part of the screen for a given period (e.g. gaze is within a 2° radius for 100 ms or longer). This can be computationally extracted from eye-tracking data using many different techniques. A common method is using the velocity signal of the gaze position. If the velocity is below a certain threshold, a sample is classified as belonging to a fixation. For more information on conceptual or computational definitions of fixations or saccades, see Hessels et al. (2018).

3 We are aware of the fact that there are developmental eye-tracking researchers who prefer not to use a car seat as infants may cry more in a car seat than e.g. on the parents lap. While our first choice is the use of a car seat to minimize infant movement, we opt for either a high chair or placing the infant on the parent's lap when the infant does not tolerate the car seat or when the parent informs the RA that the infant will not tolerate the car seat.

4 Using the *fitdist* function with the ‘kernel’-option for distribution type and the default bandwidth value.
